# Design of cinnamaldehyde amino acid Schiff base compounds based on the quantitative structure–activity relationship

**DOI:** 10.1098/rsos.170516

**Published:** 2017-09-06

**Authors:** Hui Wang, Mingyue Jiang, Shujun Li, Chung-Yun Hse, Chunde Jin, Fangli Sun, Zhuo Li

**Affiliations:** 1 Key Laboratory of Bio-Based Material Science and Technology of the Ministry of Education, Northeast Forestry University, Harbin 150040, People's Republic of China; 2 Southern Research Station, USDA Forest Service, Pineville, LA 71360, USA; 3 Key Laboratory of Wood Science and Technology, Zhejiang Agriculture and Forestry University, Zhejiang 311300, People's Republic of China

**Keywords:** cinnamaldehyde, quantitative structure–activity relationship, Schiff base, antifungal activity

## Abstract

Cinnamaldehyde amino acid Schiff base (CAAS) is a new class of safe, bioactive compounds which could be developed as potential antifungal agents for fungal infections. To design new cinnamaldehyde amino acid Schiff base compounds with high bioactivity, the quantitative structure–activity relationships (QSARs) for CAAS compounds against *Aspergillus niger* (*A. niger*) and *Penicillium citrinum (P. citrinum)* were analysed. The QSAR models (*R*^2^ = 0.9346 for *A. niger*, *R*^2^ = 0.9590 for *P. citrinum,*) were constructed and validated. The models indicated that the molecular polarity and the Max atomic orbital electronic population had a significant effect on antifungal activity. Based on the best QSAR models, two new compounds were designed and synthesized. Antifungal activity tests proved that both of them have great bioactivity against the selected fungi.

## 1. Introduction

Primary and opportunistic antifungal infections are a severe threat to human life and health [1]. As fungal resistance increases, many antifungal compounds have become ineffective [2]. It is therefore necessary to explore new, novel antifungal formulations to control fungal infections [3]. Natural products and modified natural-derived compounds have continued to play a highly significant role in the discovery of antifungal agents [4]. Researchers have modified natural, antifungal compounds to meet key requirements for practical applications. Cinnamon oil is a natural, antifungal substance and its main component is cinnamaldehyde [5]. Numerous studies have reported that cinnamaldehyde could inhibit the growth of the pathogenic microorganisms *Aspergillus niger*, *Trametes versicolor* and *Staphylococcu saureus* [6]. Cinnamaldehyde also exhibited potential anti-tumour [7] and anti-diabetes [8] properties. Also, cinnamaldehyde is generally recognized as safe and is allowed as a food additive or antimicrobial agent by the US FDA (Food and Drug Administration) [9]. However, cinnamaldehyde as either an antimicrobial agent or food additive has many practical limitations largely due to its high volatility and strong odours [10].

**Figure 1.**
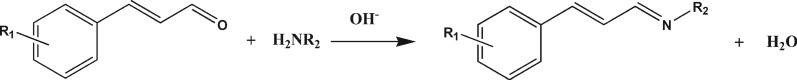
The synthesis route of cinnamaldehyde Schiff base.

Hence, many researchers have shifted their attention to cinnamaldehyde derivatives. Sharma *et al.* [11] synthesized cinnamaldehyde derivatives and cinnamaldehyde Schiff base. The results indicated that the presence of a methoxyl group on cinnamaldehyde benzene ring and cinnamaldehyde Schiff base led to a noticeable improvement in antifungal activity. Cinnamaldehyde Schiff base is an important class of cinnamaldehyde derivatives with excellent bioactivity and can be synthesized using a simple method [12]; the synthesis route is shown in figure 1. The bioactivity of cinnamaldehyde Schiff base compounds has been reported by many researchers. Zahan *et al.* [13] studied the dithiocarbazata cinnamaldehyde Schiff base compound and the metal complex compound. The bioactivity test showed that cinnamaldehyde Schiff base and metal complex exhibited comparative activity to cinnamaldehyde. Wei *et al.* [14] published a research on cinnamaldehyde amino acid Schiff base. Results indicated that Schiff base compounds were more active than the reference benzoic acid against *Bacillus subtilis, Escherichia coli* and *Saccharomyces cerevisiae*. Hence, it is meaningful to explore and design new cinnamaldehyde Schiff base compounds with favourable bioactivity. In a previous study, the antimicrobial activity of several cinnamaldehyde amino acid Schiff base compounds were studied [15]; the antimicrobial activity results implied that cinnamaldehyde amino acid Schiff base compounds possessed excellent antifungal activity, good water solubility and an odour. Cinnamaldehyde amino acid Schiff base has potential to be an antifungal agent. After an initial analysis regarding the structure and activity, its antifungal activity was found to be significantly influenced by its chemical structure. A comprehensive study on the relationship between activity and compounds should be conducted for designing the new cinnamaldehyde compounds. One approach is to design compounds using computer applications such as quantitative structure–activity relationship (QSAR) [16]. QSAR provides a mathematically quantified relationship between a molecule's structural descriptors and a compound's bioactivity at the molecular level, and can predict the activity of compounds including those not yet synthesized [17]. Using this approach, there is no need to synthesize each compound to discover those that possess the desired activity. Promising compounds can be further screened for synthesis in the laboratory.

This paper focuses on the use of QSAR for cinnamaldehyde amino acid Schiff base compounds to present a comprehensive analysis on the relationship between the bioactivity and structures of cinnamaldehyde amino acid Schiff base (CAAS) compounds. Under the guidance of QSAR models, two designed cinnamaldehyde compounds were synthesized and their antifungal activities were determined.

## 2. Material and methods

### 2.1. Materials

**Figure 2.**
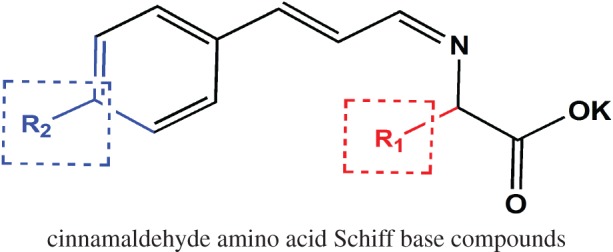
Structure of the CAAS compound used in this study.

Cinnamon oil (95% cinnamaldehyde) was provided by Zhen xing Spices Oil Refinery of Ji'an City, China. All other chemicals were analytical grade reagents and were used as received without further purification. The structure of the CAAS compound is shown in figure 2. The functional groups of CAAS compounds are listed in tables 1 and 2. Test microorganisms were two mould species provided by the Chinese Center of Industrial Culture Collection (CICC), Beijing, China. They were *A. niger* (CICC2487) and *Penicillium citrinum* (CICC4010)).

**Table 1.** The antifungal activity rate and value of descriptors for CAAS compounds against *A. niger*.

ID	R1	R2	AR	lgAR	polarity parameter/ square distance, *D*_1_	RNCG relative negative charge (QMNEG/ QTMINUS), *D*_2_	ESP-HA- dependent HDCA-1, *D*_3_	max. total interaction for a C─O bond, *D*_4_
1	−(CH_2_)_2_COOK	−H	121.41	2.0842	5.0339 × 10^−3^	0.1595	6.0067	26.8560
2	− (CH_2_)_2_COOK	−*p*-OCH_3_	108.73	2.0364	2.7696 × 10^−3^	0.2546	6.4464	26.8830
3	− (CH_2_)_2_COOK	−*p*-Cl	124.80	2.0962	2.1257 × 10^−3^	0.1624	5.2253	26.8680
4	−H	−H	46.60	1.6684	0.1249	0.2283	4.8810	26.8550
5	−H	−*p*-OCH_3_	94.80	1.9768	2.3127 × 10^−3^	0.3476	3.2558	26.8640
6	−H	−*p*-Cl	123.19	2.0906	1.6089 × 10^−3^	0.2337	3.5317	26.8760
7	−CH_3_	−H	44.62	1.6495	0.1249	0.2189	3.8374	26.8200
8	−CH_3_	−*p*-OCH_3_	56.24	1.7500	2.5943 × 10^−3^	0.3363	5.5555	26.8410
9	−CH_3_	−*p*-Cl	132.30	2.1216	1.7586 × 10^−3^	0.2246	4.3831	26.8550
10	CH_2_CH(CH_3_)_2_	−H	52.49	1.7201	0.1249	0.1726	5.1551	26.8560
11	CH_2_CH(CH_3_)_2_	−*p*-OCH_3_	85.70	1.9330	2.8601 × 10^−3^	0.2791	5.1706	26.8210
12	CH_2_CH(CH_3_)_2_	−*p*-Cl	110.87	2.0448	1.9326 × 10^−3^	0.1805	5.4321	26.8270
13	−CH(CH_3_)_2_	−H	65.88	1.8188	0.1250	0.1943	3.9828	26.8430
14	−CH(CH_3_)_2_	−*p*-OCH_3_	71.24	1.8527	3.0117 × 10^−3^	0.3069	5.8446	26.8390
15	−CH(CH_3_)_2_	−*p*-Cl	128.55	2.1091	2.0303 × 10^−3^	0.2016	3.5952	26.8300
16	−CH_2_Ar-OH	−H	100.16	2.0007	4.2908 × 10^−3^	0.2203	8.1783	26.8180
17	−CH_2_Ar-OH	−*p*-OCH_3_	78.74	1.8962	3.8575 × 10^−3^	0.2668	9.9386	26.7910
18	−CH_2_Ar-OH	−*p*-Cl	83.93	1.9239	4.2916 × 10^−3^	0.2072	10.8962	26.8320
19	−CH_2_Ar	−H	49.28	1.6926	0.1251	0.1791	5.2440	26.8050
20	−CH_2_Ar	−*p*-OCH_3_	69.63	1.8428	2.6664 × 10^−3^	0.2879	5.6060	26.8070
21	−CH_2_Ar	−*p*-Cl	105.52	2.0233	1.9517 × 10^−3^	0.1871	5.7111	26.8080

**Table 2.** The antifungal activity rates and values of descriptors for CAAS compounds against *P. citrinum*.

ID	R1	R2	AR	lgAR	max. atomic orbital electronic population, *D*_5_	max. electroph. react. index for a C atom, *D*_6_	PNSA-2 total charge weighted PNSA [Zefirov's PC], *D*_7_	max. 1-electron react. index for a O atom, *D*_8_
1	−(CH_2_)_2_COOK	−H	195.08	2.2902	1.9133	0.0229	−198.7038	3.5114 × 10^−4^
2	−(CH_2_)_2_COOK	−*p*-OCH_3_	195.08	2.2902	1.9133	0.0267	−196.8914	1.2945 × 10^−4^
3	−(CH_2_)_2_COOK	−*p*-Cl	188.69	2.2758	1.9819	0.0177	−197.1382	−3.0860 × 10^−7^
4	−H	−H	79.58	1.9008	1.9089	0.0204	−101.4046	1.0495 × 10^−5^
5	−H	−*p*-OCH_3_	89.85	1.9535	1.9089	0.0199	−130.0808	1.0946 × 10^−5^
6	−H	−*p*-Cl	164.31	2.2157	1.9919	0.0189	−127.9426	1.1607 × 10^−6^
7	−CH_3_	−H	80.87	1.9078	1.9095	0.0214	−88.7516	−3.2397 × 10^−8^
8	−CH_3_	−*p*-OCH_3_	80.87	1.9078	1.9095	0.0206	−119.6568	−1.1939 × 10^−16^
9	−CH_3_	−*p*-Cl	207.92	2.3179	1.9819	0.0222	−126.2349	−1.0264 × 10^−16^
10	CH_2_CH(CH_3_)_2_	−H	71.88	1.8566	1.9088	0.0186	−125.6587	1.4595 × 10^−5^
11	CH_2_CH(CH_3_)_2_	−*p*-OCH_3_	92.42	1.9658	1.9088	0.0199	−156.3959	−9.8043 × 10^−5^
12	CH_2_CH(CH_3_)_2_	−*p*-Cl	197.65	2.2959	1.9819	0.0179	−165.1279	−4.5030 × 10^−6^
13	−CH(CH_3_)_2_	−H	74.44	1.8718	1.9095	0.0213	−92.5453	1.5018 × 10^−7^
14	−CH(CH_3_)_2_	−*p*-OCH_3_	86.65	1.9378	1.9095	0.0227	−124.8644	1.3298 × 10^−8^
15	−CH(CH_3_)_2_	−*p*-Cl	238.77	2.3780	1.9819	0.0248	−132.3244	1.9845 × 10^−6^
16	−CH_2_Ar-OH	−H	88.57	1.9473	1.9087	0.0185	−159.8114	4.1686 × 10^−7^
17	−CH_2_Ar-OH	−*p*-OCH_3_	127.08	2.1041	1.9087	0.0161	−172.6105	1.8280 × 10^−3^
18	−CH_2_Ar-OH	−*p*-Cl	181.00	2.2577	1.9819	0.0189	−176.7350	1.8744 × 10^−4^
19	−CH_2_Ar	−H	74.45	1.8718	1.9096	0.0205	−131.3740	6.5028 × 10^−6^
20	−CH_2_Ar	−*p*-OCH_3_	151.46	2.1803	1.9096	0.0194	−179.6525	2.3434 × 10^−3^
21	−CH_2_Ar	−*p*-Cl	178.42	2.2514	1.9819	0.0172	−168.2581	1.6235 × 10^−6^

### 2.2. Determination of antifungal activity

The antifungal activity of all CAAS compounds was determined according to the Paper Disc Method against *A. niger* and *P. citrinum*. In brief, the procedure is as follows. Potato dextrose agar (PDA) medium with 2% agar was prepared and sterilized for use. The sterilized Petri dishes and PDA medium were sterilized under UV-irradiation for 20 min. The strain suspension was molten medium which was thoroughly mixed and then poured into the Petri dishes and allowed to solidify. The autoclaved discs (approx. 8 mm) were dipped into the test solution (concentration: 0.125 mol l^−1^) for 10 min. After that, the discs were put onto the surface of the solid media strain suspension mixture. The test samples were cultured at 28°C for 2 days. All tests were carried out in triplicate and the diameter of the inhibition zone was the average of those of the three test zones.

In this experiment, a well-known commercial antifungal compound fluconazole served as control. The antifungal activity rate (AR) was calculated using the followed equation:

2.1 $$\text{AR}=\left(\frac{\text{d}T}{\text{d}C}\right)\times 100\%,$$

where d*T* and d*C* were the diameter of the inhibition zone for the test compounds and fluconazole, respectively. The antifungal activity rates (ARs) and the log_10_AR (lgAR) of all CAAS compounds are listed in tables 1 and 2. The lgAR was used to compute the relationship between antifungal activity and structure of cinnamaldehyde compounds. The values of d*C* for the control compound fluconazole were 18.7 mm and 13 mm against *A. niger* and *P. citrinum*, respectively.

### 2.3. The method used for quantitative structure–activity relationship calculations

The three-dimensional structures of the compounds were drawn using the Chembio 3D 12.0 software, and the chemical structures were imported for geometrical optimization using the AMPAC Agui 9.2.1 software [18]. Secondly, the output file of the 21 compounds’ structural information and the lgAR were imported in the CODESSA 2.7.16 software to compute molecular descriptors.

Then, a ‘best multilinear regression’ function was conducted in the CODESSA 2.7.16 software to calculate the regression relationship between chemical structures and antifungal activity. Then, the number of descriptors and the optimal QSAR models were determined by the ‘breaking point’ rule [19] (The squared correlation (*R*^2^) of the model dramatically increased over the number of descriptors, but after a certain point, the increase is less significant [20]. That point is the ‘breaking point’.) To analyse the descriptor, charge distribution and density of cinnamaldehyde compounds were calculated by the Gaussian 09 and Gauss View 5.0 software for further analysis of molecular descriptors. The calculation was performed in ground state/DFT/B3LYP/3-21G.

### 2.4. The validation of optimal models

Model validation was conducted using a process of internal validation and ‘leave one out’ cross-validation [21]. In short, 21 compounds were classified into three small groups termed a, b and c with seven compounds in each group. Each of two small groups were combined as the training set A(a + b), B(a + c), C(b + c), and the correspondingly remaining groups c, b and a were considered the test set. Using the same descriptors of the best model, ‘multilinear regression’ was conducted to obtain a regression model for the training set and this model was used to predict the lgAR of the test set. The statistical results, correlation coefficient (*R*^2^), Fisher value (*F*) and standard deviation (*s*^2^) are listed in table 4. The ‘leave one out’ cross-validation is similar to the internal validation, which was conducted as follows. Every fourth compound was set as the external test set d(4, 8, 12, 16 and 20) and the other compounds were considered to be the training set *D*. Similarly, a training set model was obtained by computing the multilinear regression with the same descriptors of the best model. The obtained training set model was used to predict the corresponding external test set.

## 3. Results and discussion

### 3.1. Establishing the optimal quantitative structure–activity relationship models

**Figure 3.**
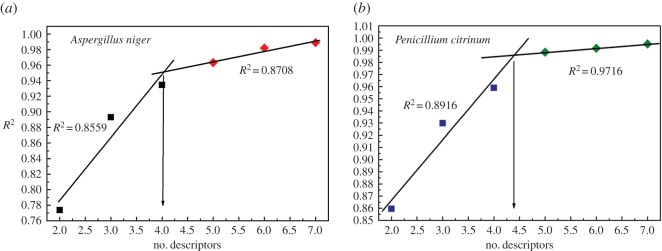
Breaking point rule for determination of the descriptor number ((*a*) *A. niger* and (*b*) *P. citrinum*).

A series of QSAR models were obtained after performing the ‘best multilinear regression’ procedure. The optimal model was determined by a rule called ‘breaking point’ shown in figure 3, which was implemented by analysing the plot of the descriptors of the obtained models versus squared correlation corresponding to those models. In figure 3, the squared coefficient increased rapidly until the point corresponding to four descriptors. After this point, the increase in value of the squared coefficient was not as great. Hence, this point is the ‘breaking point’, and the QSAR model corresponding to the breaking point is regarded as the optimal QSAR model. Additionally, the number of descriptors should meet the requirement of multilinear regression:

3.1 N≥3(D+1),

where *N* is the sample number (21) and the *D* is descriptor number of the final QSAR models [22]. Therefore, the optimal QSAR models were selected using four descriptors. The value of each descriptor of the optimal models is listed in tables 1 and 2. These four descriptor parameters and statistical data corresponding to the optimal QSAR models were listed in table 3, and the definition and analysis for each descriptor parameter are presented in Results and discussion section.

**Table 3.** The optimal QSAR model obtained for CAAS compounds against *A. niger* and *P. citrinum*.

no	X	ΔX	*t*-test value	name of descriptor
	*A. niger* model*: R*^2^= 0.9346, *F* = 57.20 and *s*^2^ = 0.0020			
0	−3.2190 × 10^1^	1.0892 × 10^1^	−2.9555	Intercept
1	−2.9745	2.1076 × 10^−1^	−14.1132	polarity parameter/square distance, *D*_1_
2	−1.5306	1.9735 × 10^−1^	−7.7556	RNCG relative negative charge (QMNEG/QTMINUS), *D*_2_
3	−3.2064 × 10^−2^	7.8686 × 10^−3^	−4.0749	ESP-HA-dependent HDCA-1[Quantum-Chemical], *D*_3_
4	−1.2940	4.0540 × 10^−1^	3.1918	max. total interaction for a C─O bond, *D*_4_
	*P. citrinum* model: *R*^2^= 0.9590, *F* = 93.47 and *s*^2^ = 0.0018			
0	−7.2473	6.1451 × 10^−1^	−11.7937	Intercept
1	4.3411	3.1123 × 10^−1^	13.9486	max. atomic orbital electronic population, *D*_5_
2	3.0016 × 10^1^	3.9781	7.5454	max. electroph. React. index for a C atom, *D*_6_
3	−2.1543 × 10^−3^	3.2208 × 10^−4^	−6.6889	PNSA-2 total charge weighted PNSA, *D*_7_
4	8.7623 × 10^1^	1.8233 × 10^1^	4.8056	max. 1-electron react. index for a O atom, *D*_8_

**Figure 4.**
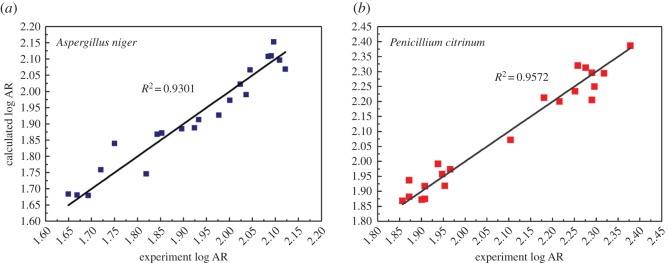
Experimental versus predicted according to the optimal models. ((*a*) *A. niger* and (*b*) *P. citrinum*).

According to the statistical data of the optimal models, the optimal QSAR models were described as fit multilinear regression equations (3.2) and (3.3). In the equations, descriptor parameters (*D*) are the independent variables, and the lgAR is the calculated value of compounds. For CAAS compounds, the predicted value (lgAR_calc_) was calculated according to the above equation and the relationships between experimental value (lgAR_exp_) and predicted value (lgAR_calc_) are presented for *A. niger* and *P. citrinum*, respectively (figure 4). In figure 4, the lgAR_exp_ and lgAR_calc_ fit in a line *y* = *x*, with *R*^2^ of 0.9572 and 0.9301 against *A. niger* and *P. citrinum*, respectively, which implied that the best QSAR models possessed good predictability. In table 4, all the validation results are satisfactory. The average of the statistical results was very close to the best model.

3.2 $$\begin{array}{rl} {\text{lgAR}}_{A.n} & =-(3.2190\pm 1.0892)\times 10^{+1}-(2.9745\pm 2.1076\times 10^{-1})\times D_{1}-(1.5306\pm 1.9735\times 10^{-1})\times D_{2}\\ & \;-(3.2064\times 10^{-2}\pm 7.8686\times 10^{-3})\times D_{3}+(1.2940\pm 4.0540\times 10^{-1})\times D_{4} \end{array}$$

and

3.3 $$\begin{array}{rl} {\text{lgAR}}_{P.c} & =-(7.2473\pm 6.1451\times 10^{-1})+(4.3411\pm 3.1123\times 10^{-1})\times D_{5}+(3.0016\times 10^{1}\pm 3.9781)\times D_{6}\\ & \;-(2.1543\times 10^{-3}\pm 3.2208\times 10^{-4})\times D_{7}+(8.7623\pm 1.8233)\times 10^{1}\times D_{8}. \end{array}$$

**Table 4.** Validation of the QSAR models for *A. niger* and *P. citrinum*.

training set	*N*	*R*^2^(fit)	*F*(fit)	*s*^2^(fit)	test set	*N*	*R*^2^(pred)	*F*(pred)	*s*^2^(pred)
	validation for the model of *A. niger*								
a + b	14	0.9410	35.88	0.0023	c	7	0.8692	39.84	0.0207
a + c	14	0.9678	67.68	0.0013	b	7	0.8629	37.75	0.0281
b + c	14	0.9367	33.31	0.0020	a	7	0.9283	77.73	0.019
average	14	0.9485	45.62	0.0019	average	7	0.8868	51.77	0.0226
*D*	16	0.9509	53.21	0.0016	d	5	0.8770	28.53	0.0252
	validation for the model of *P. citrinum*								
a + b	14	0.9623	57.39	0.0021	c	7	0.9485	110.52	0.0156
a + c	14	0.9600	53.94	0.0021	b	7	0.9334	84.05	0.0188
b + c	14	0.9711	75.54	0.0014	a	7	0.9235	72.43	0.0224
average	14	0.9645	62.29	0.0019	average	7	0.9351	89.00	0.0189
*D*	16	0.9606	66.97	0.0020	d	5	0.8934	33.53	0.0264

### 3.2. Descriptor parameters analysis for the optimal quantitative structure–activity relationship model

**Figure 5.**
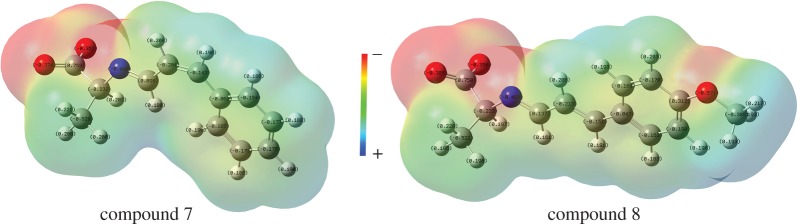
The molecular electronic potential map of compounds 7 and 8 from Gaussian 09 W calculation.

Some important structure information significantly influenced the antifungal activity involved in the optimal QSAR model. According to the optimal model listed in table 3 for the CAAS compounds against *A. niger*, there were four structural descriptors that apparently affected the antifungal activity of the CAAS compounds. The most statistically significant descriptor was the polarity parameter/square distance, *D*_1_. This is an electrostatic descriptor defined by the following equation [23,24]:

3.4 $$P^{\prime \prime }=\frac{Q_{\max}-Q_{\min}}{R_{\mathrm{mm}}^{2}},$$

where *Q*_min_ and *Q*_max_ are the most negative and the most positive atomic partial charges in the molecule, respectively, and *R*_mm_ is the distance between the most positive and the most negative atomic partial charges in the molecule. The polarity parameter reflects the polarity and characteristics of the charge distribution of the molecule. A compound with proper polarity can smoothly penetrate a fungal cell wall or cell membrane and interact with an active target. A change was observed on the value of *P*′′ when the cinnamaldehyde compounds’ structure changed, for example compound 7 had a *P*′′ value of 0.1249. The charge distribution changed when –OCH_3_ was introduced on the benzene ring (figure 5). This charge distribution led to an increase in the value of *Q*_min_ and a decrease in *P*′′ (2.5943 × 10^−3^).

**Figure 6.**
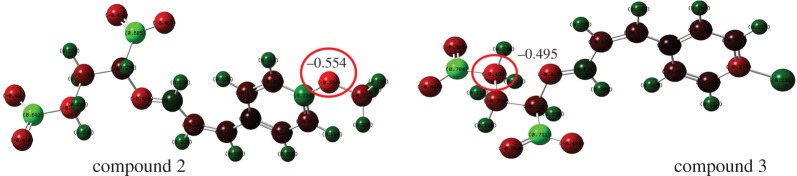
Charge distribution of compound 2 and 3 (red colour denotes a negative charge; blue colour represents a positive charge).

The second descriptor is relative negative charge (RNCG) (*D*_2_), and it is a quantum-chemical descriptor. RNCG is defined as the most negative charge divided by the total negative charge [25]:

3.5 $$\text{RNCG}=\frac{Q_{\max}^{-}}{Q^{-}},$$

where $Q_{\max}^{-}$ is the most negative charge and *Q*^−^ is the total negative charge. As shown in table 3, RNCG negatively contributed to the AR against *A. niger*. Like compounds 2, 5, 8, 11, 14, 17 and 20 had a methoxyl group (electron-donating group) on the benzene ring and resulted in an obvious increase in the descriptor RNCG compared with compounds that do not have a substituent group on the benzene ring (1, 4, 7, 10, 13, 16 and 19). Conversely, an electron-withdrawing group such as a halogen atom (chlorine) may decrease the value of *D*_2_, because different substituent groups changed the charge distribution of cinnamaldehyde compounds; for instance in compounds 2 and 3, the only difference in structure is the benzene ring 4-substituent. Compound 2 has a methoxyl group and compound 3 has a chlorine atom in the p-position on the benzene ring. These differences in substituent groups lead to a lower RNCG value for compound 3 (0.1624) than for compound 2 (0.2546). The charge distribution of optimal structures of compounds 2 and 3 by Gaussian 09 could explain it (figure 6). In figure 6, the oxygen atom in the methoxyl group had the most negative charge (−0.554) in compound 2; the total negative charge and the most negative charge changed when the substituent group changed to a Cl atom. The most negative charge of compound 3 was −0.495.

The third most important descriptor is the ESP-HA-dependent HDCA-1 (H-acceptor dependent H-donor charged surface area, *D*_3_), which is a quantum-chemical descriptor [26] that represents the hydrogen bonding donor ability of the CAAS compounds [27]. The formation of hydrogen bonding of CAAS compounds is easier as HDCA-1 increases [28]. In equation (3.2), a negative coefficient for HDCA-1 demonstrates the ability of the CAAS compounds to form hydrogen bonds which might be detrimental to antifungal activity.

The last parameter is maximum total interaction for a C─O bond (*D*_4_), which is a semi-empirical descriptor that could be used to measure the bond strength between the two atoms involved [29]. A positive coefficient implied that the strength of the C─O bond had a positive contribution to antifungal activity of cinnamaldehyde compounds against *A. niger*.

According to the optimal QSAR models against *P. citrinum*, the most statistically significant descriptor was the maximum atomic orbital electronic population (*D*_5_). It is an electrostatic descriptor and an index of nucleophilicity for cinnamaldehyde compounds [30]. The positive coefficient in the model indicated that the increase in *D*_5_ denoted antifungal activity of cinnamaldehyde compounds against *P. citrinum*.

The second descriptor is maximum electrophilic reactivity index for a C atom (*D*_6_), which is a quantum-chemical descriptor [31] that reflects the electrophilic reactivity of the C atom on cinnamaldehyde compounds. For a given atomic species A, the maximum electrophilic reactivity index for an A atom was defined as [32]

3.6 $$E_{A}=\frac{\sum_{i=1}^{n_{A}}{\text{C}}_{\mathrm{LUMO},i}^{2}}{\varepsilon_{\mathrm{LUMO}}+10},$$

where ε_LUMO_ is the energy of the lowest unoccupied molecular orbital (LUMO). Here, C_LUMO,*i*_ is the *i*th orbital coefficient of atom A on LUMO. Such summation is conducted over all valence atomic orbitals *i* in atom A(*i* = l … *n*_A_). In the best QSAR model against *P. citrinum*, a positive coefficient indicates that the antifungal activity increased as the magnitude of *D*_6_ increased.

The third descriptor parameter is PNSA-2 total charge weighted PNSA (*D*_7_). This descriptor is defined as the total negative charge multiplied by partial negative solvent-accessible surface area, which indicates the influence of negative charge distribution on the antifungal activity of cinnamaldehyde compounds [33].

The last descriptor is the maximum 1-electron reactivity index for an O atom (*D*_8_), which is a quantum-chemical descriptor [34]. It is an important descriptor parameter selected by ‘best multilinear regression’ from about 400 descriptors. In equation (3.3), *D*_8_ had a positive coefficient showing that increase in the magnitude of *D*_8_ will increase the antifungal activity of the cinnamaldehyde compound against *P. citrinum*.

## 4. Design of new compounds

According to analysis results of the two best QSAR models, the most important factors for antifungal activity were the polarity parameter (*D*_1_) and the maximum atomic orbital electronic population (*D*_5_) against *A. niger* and *P. citrinum*, respectively. For the most important structural parameter of the QSAR model against *A. niger*, some special structural factors like the number of COO^−^ and substituent groups on the benzene ring significantly decreased the value of the polarity parameter, and this decrease was very beneficial to increase antifungal activity. Hence, these special structural factors were chosen as the structural characteristics on the new design compounds. In addition, the number of COO^−^ groups of cinnamaldehyde Schiff base compounds will change the polarity of the cinnamaldehyde compounds; that is the increase in COO^−^ groups will result in an increase in the polarity of the compounds, which is beneficial to increase the water solubility of cinnamaldehyde compounds and enlarge their applied field.

With regard to the most important structural parameter of the QSAR model against *P. citrinum*, the value of *D*_5_ of the cinnamaldehyde compound was positive, contributing to the antifungal activity against *P. citrinum*. The structural characteristics like the number of COO^−^ groups and halogen atoms increased the value of *D*_5_ obviously. However, it is generally believed that halogenated hydrocarbon possesses high toxicity [35]. This structural characteristic was not considered in design compounds. Above all, the structural characteristics of the number of ─COO^−^ and ─OCH_3_ groups were selected as the key character factors in the design of compounds. Hence two new compounds were designed and synthesized. Structural characterization results are shown as follows.

Designed compound Da: Potassium (2*E*)-2-((Z)-3-phenylallylideneamino)succinate; C_13_H_13_K_2_NO_4_; M = 323.0; melting point: 198.5–200.5°C; ^1^H NMR (500 MHz, MeOD): δ 8.04 (d, *J* = 10 Hz, 1H, CH═N), 7.48 (d, *J* = 5 Hz, 2H, *Ph*-H), 7.30 (t, *J* = 5 Hz, 2H, *Ph*-H) 7.25 (t, *J* = 5 Hz, 1H, *Ph*-H), 7.02 (d, *J* = 15 Hz, 1H, C═CH), 6.88 (dd, *J* = 5, 15 Hz, 1H, CH═C), 2.78 (dd, *J* = 5, 15 Hz, 1H, CH─N), 2.61 (m, *J* = 5, 15, 25 Hz, 1H, CH─C), 2.32 (dd, *J* = 5, 15 Hz, 1H,CH─C); FTIR (cm^−1^): 1633 (υC═O), 1564 (υC═N, υC═C), 1491 (υC═C), 742 (υ(*Ph*─C─H)), 687 (υ(*Ph*─C─H)); MS (*m/z*): found [M + K]^+^ 362.0.

Designed compound Db: Potassium (2*E*)-2-((Z)-3-(4-methoxy-phenyl)-allylideneamino)succinate; C_14_H_13_K_2_NO_5_, M = 353.0; melting point: 185.8–190.0°C; ^1^H NMR (500 MHz, MeOD) δ 8.02 (d, *J* = 10 Hz, 1H, HC═N), 7.50 (d, *J* = 5 Hz, 2H, *Ph*-H), 7.04 (d, *J* = 20 Hz, 1H, C═CH), 6.93 (d, *J* = 10 Hz, 2H, *Ph*-H), 6.83 (dd, *J* = 10 Hz 20 Hz, 1H, CH═H), 3.81 (s, 3H, OCH_3_), 3.71 (m, 1H CH─N,), 2.14 (m, 2H, CH_2_─C); FTIR (cm^−1^): 1631 (υC═O), 1589 (υC═N), 1519 (υC═C), 820 (υ(*Ph*─C─H)); MS (*m/z*): found [M + K]^+^ 392.1.

**Figure 7.**
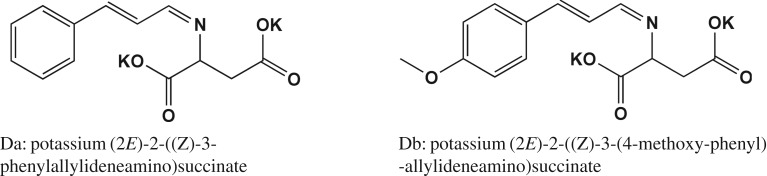
Structures of the new designed compounds.

The structures of the new designed compounds are shown in figure 7; the AR of new compounds was determined by the method described in the Material and methods section, and the results are listed in table 5.

**Table 5.** The antifungal activity rate and comparison between the predicted value and the experimental value of designed compounds Da and Db.

	no	Cal.lgAR	Exp.AR	Exp.lgAR	error
*Aspergillus niger*	Da	2.1851	128.55	2.1091	0.0760
	Db	2.0480	120.51	2.0810	−0.0330
*Penicillium citrinum*	Da	2.0275	225.92	2.3540	−0.3265
	Db	2.1125	182.28	2.2607	−0.1482

The predicted lgAR of new compounds was obtained by the following steps. First, the structures of new compounds were drawn and inputted into the AMPAC 9.21 software to geometry-optimize them and the optimized structure files were saved. Then, the optimized structure files were inputted into the CODESSA 2.7.16 software to calculate the molecular descriptors. Finally, a predict function was conducted in the condition of the best model, and the calculated value (Cal.lgAR) was obtained and listed in table 5. In table 5, the Cal.lgAR value of two compounds for both fungi were greater than that of the control compound fluconazole. The Cal.lgAR value of compound Da against *A. niger* was the greatest among those of all the compounds used for establishing the model.

The experimental results of antifungal activity have shown that new compounds exhibited better bioactivity than compounds listed in table 1. From table 5, the Exp.lgAR value was very close to that of Cal.lgAR for both new compounds against the two fungi.

The average of absolute error and the relative error were 0.0545 and 2.55% against *A. niger*, and 0.2374 and 11.55% against *P. citrinum*. These small errors implied that two best QSAR models had good predictability and were satisfactory. From another perspective, two designed compounds could be treated as the external test set to validate the best QSAR models. Small errors indicated that these two QSAR models were reliable.

## 5. Conclusion

Two QSAR models of CAAS compounds against *A. niger* and *P. citrinum* with good statistical results were obtained and validated. The definition and analysis of the important descriptor parameters implied the chemical structural characteristics which influenced antifungal activity. The results indicated that molecular polarity and negative charge distribution of cinnamaldehyde compounds were important influences on antimicrobial activity. By analysis of the descriptor parameters of these two models, some guidance was obtained on chemical structure for the design of new cinnamaldehyde compounds. Two designed compounds exhibited excellent antifungal activity against both fungi and the experimental values were very close to the predicted values. All the results indicated that two best QSAR models possessed good predictability.

## Supplementary Material

Supplementary material and original data for manuscript RSOS-170516.R1 entitled "Design of cinnamaldehyde amino acids Schiff base compounds based on the quantitative structure activity relationship".

## Data accessibility

The datasets supporting this article have been uploaded as part of the electronic supplementary material.
