# Supplementary material for: Design of cinnamaldehyde amino acid Schiff base compounds based on the quantitative structure–activity relationship
Source: R Soc Open Sci. 2017 Sep 6;4(9):170516. doi: 10.1098/rsos.170516 (PMC5627098; doi:10.1098/rsos.170516)
Supplement: Supplementary material and original data for manuscript RSOS-170516.R1 entitled "Design of cinnamaldehyde amino acids Schiff base compounds based on the quantitative structure activity relationship". [file rsos170516supp1.docx]

**Supplementary Information**

**Journal:** Royal Society Open Science

**Title:** Design of cinnamaldehyde amino acids Schiff base compounds based on the quantitative structure activity relationship

**Authors:**

Hui Wang^1, 2^, Mingyue Jiang^1^, Shujun Li^1, ^[[1]](#footnote-1)^*^, Chung-Yun Hse^2^, Chunde Jin^3^, Fangli Sun^3^ and Zhuo Li^1^

**Affiliations:**

^1^Key Laboratory of Bio-Based Material Science and Technology of the Ministry of Education, Northeast Forestry University, Harbin 150040, China

^2^ Southern Research Station, USDA Forest Service, Pineville, Louisiana 71360, United States

^3^ Key Laboratory of Wood Science and Technology, Zhejiang agriculture and forestry University, Zhejiang 311300, China

*** Corresponding author:**

Shujun Li, Tel.& fax: +86-451-8219-1742; E-mail:lishujun@nefu.edu.cn

**Supplementary information link text:** <https://doi.org/10.6084/m9.figshare.5202496>

**Table of contents:**

Table S1. The antifungal activity of all compounds against *Aspergillus niger* 2

Table S2. The antifungal activity of all compounds against *Penicillium citrinum* 3

Table S3. The Experimental lgAR versus Calculated lgAR according to the best QSAR model against *Aspergillus niger* 4

Table S4. The Experimental lgAR versus Calculated lgAR according to the best QSAR model against *Penicillium citrinum* 5

Table S5. Grouping results for validation of QSAR model against *Aspergillus niger* and *Penicillium citrinum* 6

Table S6. The validation results of best QSAR models against *Aspergillus niger*  7

Table S7. The validation results of best QSAR models against *Penicillium citrinum* 8

The synthesis method of new designed compounds 9

The structures of two designed compounds 10

^1^H NMR, IR and MS spectra of compound Da 11-12

^1^H NMR, IR and MS spectra of compound Db 13-14

| **Table S1. Antifungal activity rates of all compounds against *Aspergillus niger*** | | | | |
| --- | --- | --- | --- | --- |
| **No.** | **Diameter of inhibition zone(dT)** | **dT/Dc** | **Antifungal activity(AR)** | **lgAR** |
| 1 | 22.67 | 1.2141 | 121.4071 | 2.0842 |
| 2 | 20.30 | 1.0873 | 108.7306 | 2.0364 |
| 3 | 23.30 | 1.2480 | 124.7991 | 2.0962 |
| 4 | 8.70 | 0.4660 | 46.5988 | 1.6684 |
| 5 | 17.70 | 0.9480 | 94.8045 | 1.9768 |
| 6 | 23.00 | 1.2319 | 123.1923 | 2.0906 |
| 7 | 8.33 | 0.4462 | 44.6170 | 1.6495 |
| 8 | 10.50 | 0.5624 | 56.2400 | 1.7500 |
| 9 | 24.70 | 1.3230 | 132.2978 | 2.1216 |
| 10 | 9.80 | 0.5249 | 52.4906 | 1.7201 |
| 11 | 16.00 | 0.8570 | 85.6990 | 1.9330 |
| 12 | 20.70 | 1.1087 | 110.8731 | 2.0448 |
| 13 | 12.30 | 0.6588 | 65.8811 | 1.8188 |
| 14 | 13.30 | 0.7124 | 71.2373 | 1.8527 |
| 15 | 24.00 | 1.2855 | 128.5485 | 2.1091 |
| 16 | 18.70 | 1.0016 | 100.1607 | 2.0007 |
| 17 | 14.70 | 0.7874 | 78.7359 | 1.8962 |
| 18 | 15.67 | 0.8393 | 83.9314 | 1.9239 |
| 19 | 9.20 | 0.4928 | 49.2769 | 1.6926 |
| 20 | 13.00 | 0.6963 | 69.6304 | 1.8428 |
| 21 | 19.70 | 1.0552 | 105.5169 | 2.0233 |
| Da | 24.00 | 1.2855 | 128.5485 | 2.1091 |
| Db | 22.50 | 1.2051 | 120.5142 | 2.0810 |

Note: The antifungal activities of all the compounds were determined by paper disc method. The test concentration was 0.125mol/L. The antifungal activity rates were calculated as following equation: AR= (dT/dC) ×100%

dC was the diameter of inhibition zone of The values of dC for control compound-fluconazole, the values were 18.7mm against *A. niger*. lgAR used for calculating the QSAR model by Codessa 2.7.16 software.

| **Table S2. Antifungal activity rates of all compounds against *Penicillium citrinum*** | | | | |
| --- | --- | --- | --- | --- |
| **No.** | **Diameter of inhibition zone(dT)** | **dT/Dc** | **Antifungal activity(AR)** | **lgAR** |
| 1 | 25.36 | 1.9508 | 195.08 | 2.2902 |
| 2 | 25.36 | 1.9508 | 195.08 | 2.2902 |
| 3 | 24.53 | 1.8869 | 188.69 | 2.2758 |
| 4 | 10.35 | 0.7958 | 79.58 | 1.9008 |
| 5 | 11.68 | 0.8985 | 89.85 | 1.9535 |
| 6 | 21.36 | 1.6431 | 164.31 | 2.2157 |
| 7 | 10.51 | 0.8087 | 80.87 | 1.9078 |
| 8 | 10.51 | 0.8087 | 80.87 | 1.9078 |
| 9 | 27.03 | 2.0792 | 207.92 | 2.3179 |
| 10 | 9.35 | 0.7188 | 71.88 | 1.8566 |
| 11 | 12.02 | 0.9242 | 92.42 | 1.9658 |
| 12 | 25.70 | 1.9765 | 197.65 | 2.2959 |
| 13 | 9.68 | 0.7444 | 74.44 | 1.8718 |
| 14 | 11.27 | 0.8665 | 86.65 | 1.9378 |
| 15 | 31.04 | 2.3877 | 238.77 | 2.3780 |
| 16 | 11.51 | 0.8857 | 88.57 | 1.9473 |
| 17 | 16.52 | 1.2708 | 127.08 | 2.1041 |
| 18 | 23.53 | 1.8100 | 181.00 | 2.2577 |
| 19 | 9.68 | 0.7445 | 74.45 | 1.8718 |
| 20 | 19.69 | 1.5146 | 151.46 | 2.1803 |
| 21 | 23.19 | 1.7842 | 178.42 | 2.2514 |
| Da | 29.37 | 2.2592 | 225.92 | 2.3540 |
| Db | 23.696 | 1.8228 | 182.28 | 2.2607 |

Note: The antifungal activities of all the compounds were determined by paper disc method. The test concentration was 0.125mol/L. The antifungal activity rates were calculated as following equation: AR= (dT/dC) × 100%

dC was the diameter of inhibition zone of The values of dC for control compound-fluconazole, the values were 13mm against *P. citrinum*, respectively. lgAR used for calculating the QSAR model by Codessa 2.7.16 software.

**Table S3 The Experimental lgAR versus Calculated lgAR according to the best QSAR model against *Aspergillus niger***

| **No.** | **Experimental lgAR** | **Calculated lgAR** | **Error** |
| --- | --- | --- | --- |
| 1 | 2.0842 | 2.1082 | -0.024 |
| 2 | 2.0364 | 1.9903 | 0.0461 |
| 3 | 2.0962 | 2.1531 | -0.0569 |
| 4 | 1.6684 | 1.6811 | -0.0127 |
| 5 | 1.9768 | 1.927 | 0.0498 |
| 6 | 2.0906 | 2.1101 | -0.0195 |
| 7 | 1.6495 | 1.6839 | -0.0344 |
| 8 | 1.75 | 1.8399 | -0.0899 |
| 9 | 2.1216 | 2.069 | 0.0526 |
| 10 | 1.7201 | 1.7588 | -0.0387 |
| 11 | 1.933 | 1.9132 | 0.0198 |
| 12 | 2.0448 | 2.0663 | -0.0215 |
| 13 | 1.8188 | 1.7462 | 0.0726 |
| 14 | 1.8527 | 1.8718 | -0.0191 |
| 15 | 2.1091 | 2.0964 | 0.0127 |
| 16 | 2.0007 | 1.973 | 0.0277 |
| 17 | 1.8962 | 1.8851 | 0.0111 |
| 18 | 1.9239 | 1.8879 | 0.036 |
| 19 | 1.6926 | 1.6796 | 0.013 |
| 20 | 1.8428 | 1.8681 | -0.0253 |
| 21 | 2.0233 | 2.0225 | 0.0008 |

NOTE: The calculated lgAR were calculated by best QSAR model, and the difference between calculated lgAR and experimental lgAR indicated the predictability of the QSAR model. The above results processed by Orign 8.0 software and corresponded to Figure 4 in manuscript.

**Table S4 The Experimental lgAR versus Calculated lgAR according to the best QSAR model against *Penicillium citrinum***

| **No.** | **Expmental lgAR** | **Calculated lgAR** | **Error** |
| --- | --- | --- | --- |
| 1 | 2.2902 | 2.2051 | 0.0851 |
| 2 | 2.2902 | 2.2959 | -0.0057 |
| 3 | 2.2758 | 2.3129 | -0.0371 |
| 4 | 1.9008 | 1.8724 | 0.0284 |
| 5 | 1.9535 | 1.9184 | 0.0351 |
| 6 | 2.2157 | 2.2003 | 0.0154 |
| 7 | 1.9078 | 1.875 | 0.0328 |
| 8 | 1.9078 | 1.9172 | -0.0094 |
| 9 | 2.3179 | 2.2945 | 0.0234 |
| 10 | 1.8566 | 1.8685 | -0.0119 |
| 11 | 1.9658 | 1.9738 | -0.008 |
| 12 | 2.2959 | 2.2499 | 0.046 |
| 13 | 1.8718 | 1.8823 | -0.0105 |
| 14 | 1.9378 | 1.992 | -0.0542 |
| 15 | 2.378 | 2.3865 | -0.0085 |
| 16 | 1.9473 | 1.9577 | -0.0104 |
| 17 | 2.1041 | 2.072 | 0.0321 |
| 18 | 2.2577 | 2.3201 | -0.0624 |
| 19 | 1.8718 | 1.937 | -0.0652 |
| 20 | 2.1803 | 2.2128 | -0.0325 |
| 21 | 2.2514 | 2.2341 | 0.0173 |

NOTE: The calculated lgAR were calculated by best QSAR model, and the difference between calculated lgAR and experimental lgAR indicated the predictability of the QSAR model. The above results processed by Orign 8.0 software and corresponded to Figure 4 in manuscript.

**Table S5 Grouping results for validation of QSAR model against *Aspergillus niger* and *Penicillium citrinum***

| **group a** | 1, 6, 8, 10, 15, 17, 19 |
| --- | --- |
| **group b** | 2, 4, 9, 11, 13, 18, 20 |
| **group c** | 3, 5, 7, 12, 14, 16, 21 |
| **group d** | 4, 8, 12, 16, 20 |
| **group A(a+b)** | 1, 2, 4, 6, 8, 9, 10, 11, 13, 15, 17, 18, 19, 20 |
| **group B(a+c)** | 1, 3, 5, 6, 7, 8, 10, 12, 14, 15, 16, 17, 19, 21 |
| **group C(b+c)** | 2, 3, 4, 5, 7, 9, 11, 12, 13, 14, 16, 18, 20, 21 |
| **group D** | 1, 2, 3, 5, 6, 7, 9, 10, 11, 13, 14, 15, 17, 18, 19, 21 |

Note: The grouping details and validation results are listed in following Tables, The validation results processed by Orign 8.0 software. Following results were corresponded to Table 4 in manuscript.

**Table S6 The validation results of best QSAR models against *Aspergillus niger***

| Using group A to predict group c, the results are as follows: | | |
| --- | --- | --- |
| **No.** | **Predicted lgAR** | **Experimental lgAR** |
| 3 | 2.1809 | 2.0962 |
| 5 | 1.8887 | 1.9768 |
| 7 | 1.6849 | 1.6495 |
| 12 | 2.0925 | 2.0448 |
| 14 | 1.8523 | 1.8527 |
| 16 | 1.9863 | 2.0007 |
| 21 | 2.0487 | 2.0233 |
| Using group B to predict group b, the results are as follows: | | |
| **No.** | **Predicted lgAR** | **Experimental lgAR** |
| 2 | 1.9184 | 2.0364 |
| 4 | 1.6365 | 1.6684 |
| 9 | 2.0578 | 2.1216 |
| 11 | 1.9094 | 1.933 |
| 13 | 1.7287 | 1.8188 |
| 18 | 1.7941 | 1.9239 |
| 20 | 1.8661 | 1.8428 |
| Using group C to predict group a, the results are as follows: | | |
| **No.** | **Predicted lgAR** | **Experimental lgAR** |
| 1 | 2.113 | 2.0842 |
| 6 | 2.1275 | 2.0906 |
| 8 | 1.8664 | 1.75 |
| 10 | 1.7705 | 1.7201 |
| 15 | 2.0893 | 2.1091 |
| 17 | 1.8782 | 1.8962 |
| 19 | 1.6712 | 1.6926 |
| Using group D to predict group d, the results are as follows: | | |
| **No.** | **Predicted lgAR** | **Experimental lgAR** |
| 4 | 1.6963 | 1.6684 |
| 8 | 1.8793 | 1.75 |
| 12 | 2.0591 | 2.0448 |
| 16 | 1.9792 | 2.0007 |
| 20 | 1.8932 | 1.8428 |

**Table S8 The validation results of best QSAR models against *Penicillium citrinum***

| Using group A to predict group c, the results are as follows: | | |
| --- | --- | --- |
| **No.** | **Predicted lgAR** | **Experimental lgAR** |
| 3 | 2.3052 | 2.2758 |
| 5 | 1.9146 | 1.9535 |
| 7 | 1.8699 | 1.9078 |
| 12 | 2.2395 | 2.2959 |
| 14 | 1.9929 | 1.9378 |
| 16 | 1.9539 | 1.9473 |
| 21 | 2.2226 | 2.2514 |
| Using group B to predict group b, the results are as follows: | | |
| **No.** | **Predicted lgAR** | **Experimental lgAR** |
| 2 | 2.3242 | 2.2902 |
| 4 | 1.8613 | 1.9008 |
| 9 | 2.2996 | 2.3179 |
| 11 | 1.9747 | 1.9658 |
| 13 | 1.8706 | 1.8718 |
| 18 | 2.3355 | 2.2577 |
| 20 | 2.2767 | 2.1803 |
| Using group C to predict group a, the results are as follows: | | |
| **No.** | **Predicted lgAR** | **Experimental lgAR** |
| 1 | 2.1763 | 2.2902 |
| 6 | 2.2130 | 2.2157 |
| 8 | 1.9238 | 1.9078 |
| 10 | 1.8745 | 1.8566 |
| 15 | 2.3917 | 2.3780 |
| 17 | 2.0474 | 2.1041 |
| 19 | 1.9390 | 1.8718 |
| Using group D to predict group d, the results are as follows: | | |
| **No.** | **Predicted lgAR** | **Experimental lgAR** |
| 4 | 1.8683 | 1.9008 |
| 8 | 1.9124 | 1.9078 |
| 12 | 2.2356 | 2.2959 |
| 16 | 1.9467 | 1.9473 |
| 20 | 2.2762 | 2.1803 |

**The synthesis method of new designed compounds**

Equal malar ratio cinnamaldehyde and aspartic acid (0.0067mol) were added into 30 mL methanol. Then, 0.0134mol potassium hydroxide was added in to 20 mL methanol to prepare the potassium hydroxide methanol solution. Under stirring, potassium hydroxide methanol solution added into cinnamaldehyde aspartic acid mixture drop by drop until aspartic acid completely dissolved. After that, keep stirring the mixture for 5 hours at room temperature. Finally, the solvent was removed by rotary evaporation at 35 **°C**. The target compounds will be gained by added 15 mL diethyl ether. Then, the precipitate was washed three times and vacuum dried for 6 hours.

**The structures of two designed compounds**

**^1^****H NMR, IR and MS spectra of compound Da**

**IR for Compound Da**

**^1^H NMR for compound Da**

**MS for Compound Da**


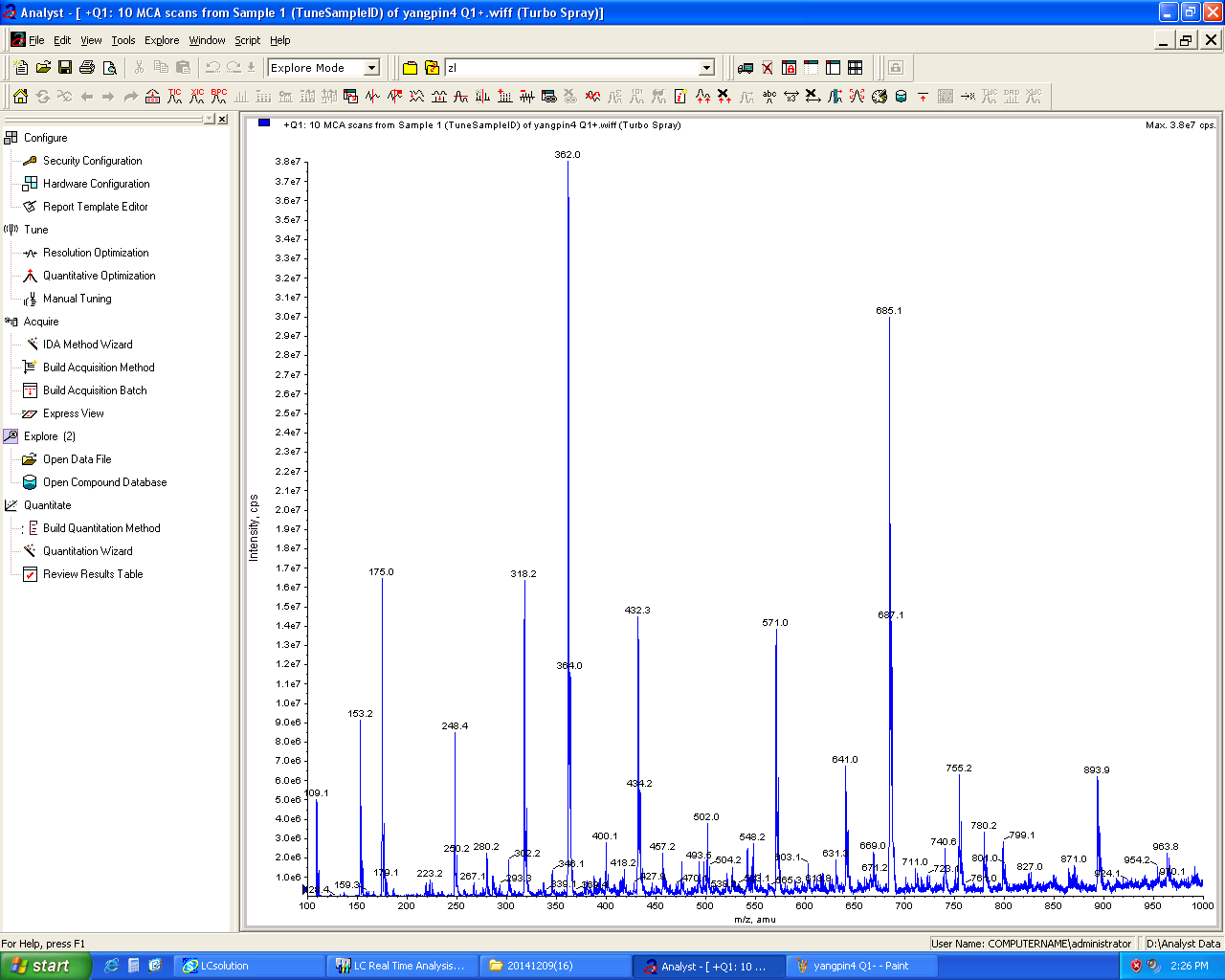


**^1^H NMR, IR and MS spectra of compound Db**

**IR for Compound Db**

**^1^H NMR for compound Db**

**MS for Compound Db**


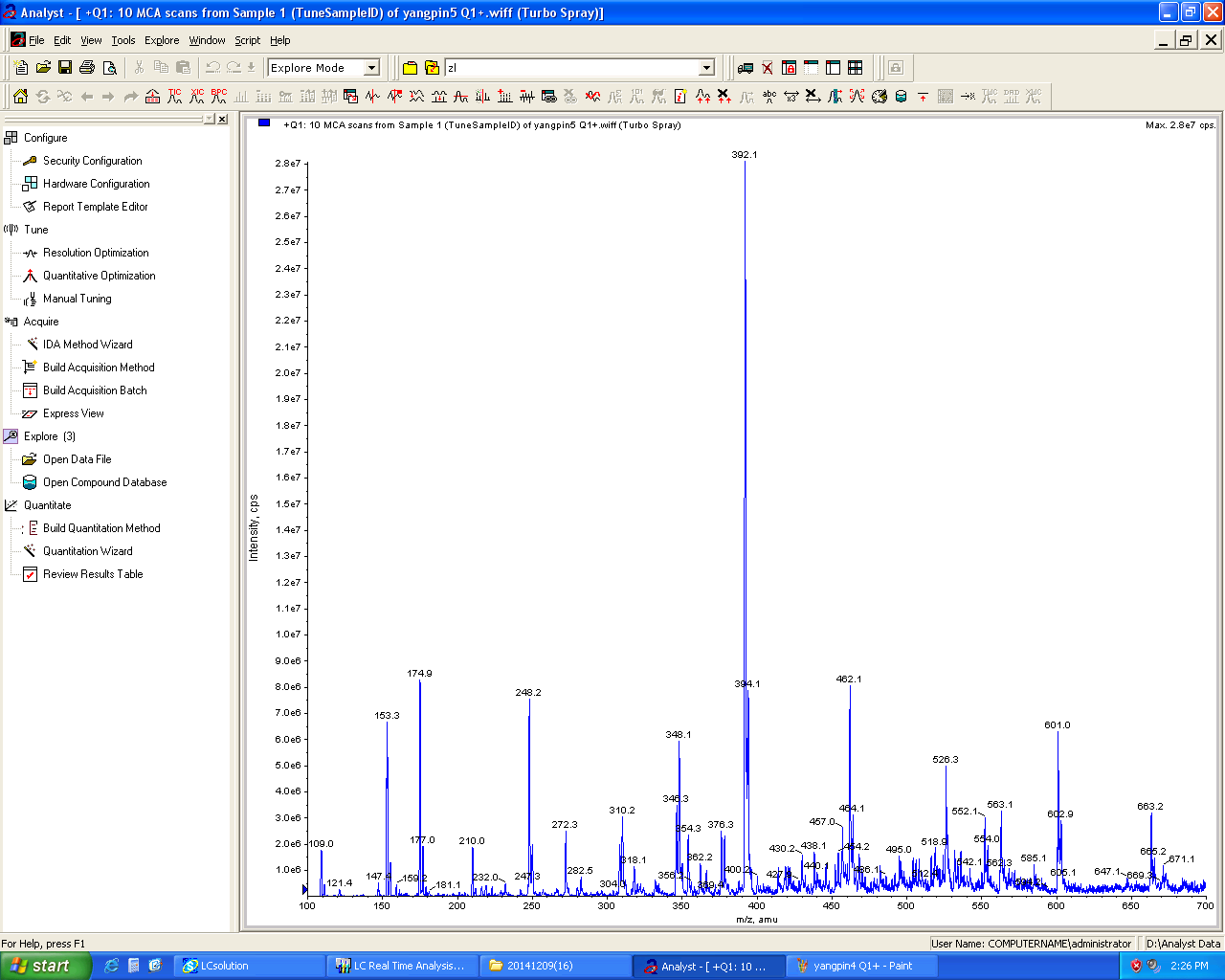


1. [↑](#footnote-ref-1)
